# Diabetes mellitus type 2 in urban Ghana: characteristics and associated factors

**DOI:** 10.1186/1471-2458-12-210

**Published:** 2012-03-20

**Authors:** Ina Danquah, George Bedu-Addo, Karl-Johann Terpe, Frank Micah, Yaw A Amoako, Yaw A Awuku, Ekkehart Dietz, Markus van der Giet, Joachim Spranger, Frank P Mockenhaupt

**Affiliations:** 1 Institute of Tropical Medicine and International Health, Charité - University Medicine Berlin, Berlin, Germany; 2 Department of Molecular Epidemiology, German Institute of Human Nutrition Potsdam-Rehbruecke, Nuthetal, Germany; 3 Komfo Anokye Teaching Hospital, Kwame Nkrumah University of Science and Technology, Kumasi, Ghana; 4 Institute of Biometry and Clinical Epidemiology, Charité - University Medicine Berlin, Berlin, Germany; 5 Department of Medicine IV - Nephrology, Charité - University Medicine Berlin, Berlin, Germany; 6 Department of Endocrinology, Diabetes and Nutritional Medicine, Charité - University Medicine Berlin, Berlin, Germany

## Abstract

**Background:**

Sub-Saharan Africa faces a rapid spread of diabetes mellitus type 2 (DM2) but its potentially specific characteristics are inadequately defined. In this hospital-based study in Kumasi, Ghana, we aimed at characterizing clinical, anthropometric, socio-economic, nutritional and behavioural parameters of DM2 patients and at identifying associated factors.

**Methods:**

Between August 2007 and June 2008, 1466 individuals were recruited from diabetes and hypertension clinics, outpatients, community, and hospital staff. Fasting plasma glucose (FPG), serum lipids and urinary albumin were measured. Physical examination, anthropometry, and interviews on medical history, socio-economic status (SES), physical activity and nutritional behaviour were performed.

**Results:**

The majority of the 675 DM2 patients (mean FPG, 8.31 mmol/L) was female (75%) and aged 40-60 years (mean, 55 years). DM2 was known in 97% of patients, almost all were on medication. Many had hypertension (63%) and microalbuminuria (43%); diabetic complications occurred in 20%. Overweight (body mass index > 25 kg/m^2^), increased body fat (> 20% (male), > 33% (female)), and central adiposity (waist-to-hip ratio > 0.90 (male), > 0.85 (female)) were frequent occurring in 53%, 56%, and 75%, respectively. Triglycerides were increased (≥ 1.695 mmol/L) in 31% and cholesterol (≥ 5.17 mmol/L) in 65%. Illiteracy (46%) was high and SES indicators generally low. Factors independently associated with DM2 included a diabetes family history (adjusted odds ratio (aOR), 3.8; 95% confidence interval (95%CI), 2.6-5.5), abdominal adiposity (aOR, 2.6; 95%CI, 1.8-3.9), increased triglycerides (aOR, 1.8; 95%CI, 1.1-3.0), and also several indicators of low SES.

**Conclusions:**

In this study from urban Ghana, DM2 affects predominantly obese patients of rather low socio-economic status and frequently is accompanied by hypertension and hyperlipidaemia. Prevention and management need to account for a specific risk profile in this population.

## Background

In sub-Saharan Africa (SSA), growth rates of diabetes mellitus (DM) and hypertension are among the highest worldwide. While today an overall DM prevalence of 4% is assumed, the number of affected patients is projected to double from 12 to 24 million within the next 20 years [1-4].

DM and other chronic diseases hit Africa in particular: The health system does not reach a considerable portion of the population, has a focus on emergencies and infectious diseases, and is frequently limited in staff and infrastructure. Not rarely, health workers are insufficiently trained in chronic disease management [2]. Severe complications and a reduced life expectancy for both diabetic and hypertensive patients are among the consequences [4-6].

In urban Ghana, type 2 DM (DM2) affects at least 6% of adults and is associated with age and obesity. Some 23% of adults are overweight, and this has been related to advanced age, female gender, urban environment, high income and tertiary education [7,8]. Epidemiological data suggest interactions between acculturation, urbanisation, and genetic disposition to be involved in DM2 among Ghanaians [5,9,10].

Contrasting increasing prevalence, severe complications and public health significance, studies on DM2 in SSA are remarkably scarce. Understanding manifestation and associated factors, however, is essential to guide diagnosis, management, and prevention of DM2 in this region. Here, we examined clinical, anthropometric, socio-economic, nutritional and behavioural parameters among 1466 urban Ghanaian adults with and without DM2 and hypertension, and present these data and an explorative analysis of associated factors in this population.

## Methods

### Study site and design

The study was conducted from August 2007 through June 2008 at Komfo Anokye Teaching Hospital (KATH) in Kumasi, Ghana. In this region, 6% and 29% of adults are affected by DM2 and hypertension, respectively [5,11,12]. At KATH, the diabetes and hypertension clinics are frequented each by > 100 patients/week. The study aimed at examining factors associated with DM2 and hypertension among hospital attendants with DM2 and/or hypertension and controls. Secondary objective was to describe the patients' clinical and biochemical characteristics. The study protocol was reviewed and approved by the Ethics Committee, School of Medicine, Kwame Nkrumah University of Science and Technology, Kumasi, and informed written consent was obtained from all participants.

### Recruitment procedures and examinations

Following study-related information, patients attending the diabetes center (*n *= 495) or the hypertension clinic (*n *= 451) were recruited. Patients encouraged members of their community to participate in the study as preliminary controls. After exclusion of DM2 and hypertension (see below), the latter were included into the study as controls (*n *= 222). Likewise, further controls were recruited among outpatients (*n *= 150) and hospital staff (*n *= 148).

From 10:00 p.m. prior to the examination day, the participants were instructed on fasting, alcohol and tobacco abstinence, and avoiding excessive physical activity. On the examination day, patients were physically examined and interviewed. Parameters assessed included: age, gender, residence, ethnic group, previous and current diseases and complaints, own and family history of DM and hypertension, medications, smoking behaviour, literacy, occupation, household size, wealth indicators, characteristics of work and recreational sports, fitness indicators as well as axillary temperature, blood pressure (0', 5', 10'; measured after resting for ten minutes; M8 Comfort, Omron, Japan), tuning fork test, and peripheral pulses and ulcers.

Fasting venous blood and urine samples were collected. Concentrations of fasting plasma glucose (FPG, fluoride plasma, +4°C) and of urinary albumin were measured photometrically (Glucose 201^+ ^Analyzer, Albumin Systems; HemoCue, Sweden). Serum triglycerides, total cholesterol and high-density lipoprotein (HDL)-cholesterol were measured by colorimetric tests (ABX Pentra400, Horiba Medical, Germany). Low-density lipoprotein (LDL) cholesterol was calculated according to the Friedewald formula [13]. If triglycerides were > 3.0 mmol/L, LDL-cholesterol was quantified directly.

### Anthropometrical examinations and nutritional interviews

Weight was measured in kg using a person scale, and height in cm by a statometer. Waist and hip circumferences were assessed in cm using a measuring tape (all devices, SECA, Germany). Body mass index (BMI) and waist-to-hip ratio (WHR) were calculated as: BMI (kg/m^2^) = weight (kg)/[height (m)]^2 ^and WHR = waist (cm)/hip (cm). Triplicates of triceps, biceps, subscapular and suprailiac skin fold thickness were measured in mm on the right-hand side of the body (Harpenden calliper, Baty International, UK). Body fat (%) was calculated according to Durnin & Womersley [14]. Also, body composition was determined under fasting conditions by bioelectric impedance analysis (BIA) and appropriate software (50 kHz, Nutrigard-S, NutriPlus 1.0; Data Input, Germany).

Daily energy expenditure was calculated as metabolic equivalents (MET) × body weight × duration of activity [15]. Trained nurses recorded weekly aliment intake with a locally-adapted food frequency questionnaire. Quantity and type of ingested foods of one day were documented by participants using a 24-hours dietary recall and handy-measured food units. Based on local and international food composition tables [16], the individual intake of macronutrients (total energy, protein, fat, carbohydrates), sodium, and fibre was calculated.

### Definitions

DM2 was defined as FPG ≥ 7 mmol/L and/or documented anti-diabetic medication [17]. Likewise, hypertension denoted a mean BP ≥ 140/90 mmHg and/or documented anti-hypertensive treatment [18]. Controls were negative for both conditions. Increased serum triglycerides were defined as ≥ 1.695 mmol/L, increased total cholesterol as ≥ 5.17 mmol/L and decreased HDL-cholesterol as ≤ 0.9 mmol/L (male) or ≤ 1.0 mmol/L (female) [17,19]. Overweight, obesity and central adiposity were classified as BMI ≥ 25.0 kg/m^2^, BMI ≥ 30.0 kg/m^2 ^and WHR > 0.90 (male) or > 0.85 (female), respectively [17]. Body fat percentage was increased at ≥ 20% (male) or ≥ 33% (female) [20].

### Statistical analysis

Assessed parameters were compared between controls and patients with diabetes, and following stratification of diabetic patients by hypertension. Between-group-comparisons of continuous parameters were done by Mann-Whitney-*U*-test and of proportions by χ^2^-test. We applied an explorative analysis, i.e., not hypothesis-driven, of factors associated with DM2 and/or hypertension. For that, all factors found to be univariately associated with e.g., DM2, were entered into a logistic regression model, *a priori *including age and gender, and odds ratios (ORs) and 95% confidence intervals (95% CI) were calculated. Stepwise backward removal of parameters loosing significant association in multivariate analysis (*P *> 0.05) identified factors associated with DM2 independently of each other. These models were applied separately for DM2, DM2 only, DM2 with hypertension, and hypertension only. Further, an alternative, fully adjusted model was established including age, gender and identified confounders.

## Results

### Recruitment and study population

Of 1,536 consenting individuals, 70 were excluded from analysis (missing FPG, 52; missing BP, 10; had eaten, 4; conflicting disease status, 4). Table 1 displays the characteristics of the remaining 1466 participants. Data stratified by gender are shown in the Additional file 1: Table S1 and Additional file 2: Table S2. The majority of the study participants was female, aged 40-60 years, of Akan ethnicity and lived in the Kumasi metropolitan area. Many had no formal education, were illiterate and unemployed. Those who worked mainly pursued light physical work. Large families (median, 3 children; range, 0-20) and households prevailed; household assets were limited. Tobacco and alcohol consumption were low (ever smoked, 6%; any intake, 15%). Overweight, obesity and abdominal adiposity, and increased body fat percentage were frequent (Table 1). The diet was rich in carbohydrates (means ± standard deviation (SD), 140 ± 40 g/4.2 MJ/d), fat (35 ± 14 g/4.2 MJ/d), and sodium (3.4 ± 1.9 g/4.2 MJ/d), moderate in protein (46 ± 14 g/4.2 MJ/d) and poor in fibre (17 ± 9 g/d). Physical activity was generally low at a mean energy expenditure of 6.3 ± 3.6 MJ/d.

**Table 1:** Characteristics of 1466 urban Ghanaians with and without type 2 diabetes mellitus and/or hypertension

*Characteristics *	Total	Controls	Diabetes mellitus type 2 (DM2)	DM2 without hypertension	DM2 with hypertension
No.	1466^a^	377	675	251	424
Age (years)	50.6 ± 15.3	38.8 ± 14.8	54.7 ± 13.4*	48.6 ± 14.0*	58.3 ± 11.6*
Sex (female, %)	75.9 (1113)	76.4 (288)	74.7 (504)	71.7 (180)	76.2 (323)
Ethnic group (Akan, %)	87.2 (1277)	84.4 (318)	87.8 (592)	83.6 (209)	90.3 (383)^†^
Residence (Kumasi metropolitan, %)	73.8 (1079)	79.3 (299)	70.8 (476)^†^	68.7 (171)^†^	72.1 (305)^†^
***Clinical data ***					
Fasting plasma glucose (mmol/l)	6.28 ± 3.52	4.5 ± 0.7	8.28 ± 4.33*	9.0 ± 4.6*	7.92 ± 4.11*
Systolic blood pressure (mmHg)	134.5 ± 23.2	116.0 ± 11.3	138.3 ± 23.9*	118.6 ± 12.6^†^	149.8 ± 21.3*
Diastolic blood pressure (mmHg)	84.8 ± 12.6	76.0 ± 7.5	85.1 ± 12.1*	76.5 ± 7.4	90.2 ± 11.4*
Triglycerides (mmol/l)	1.44 ± 0.74	1.15 ± 0.56	1.55 ± 0.81*	1.40 ± 0.64*	1.65 ± 0.88*
Total cholesterol (mmol/l)	6.08 ± 1.75	5.73 ± 1.61	5.95 ± 1.71	5.63 ± 1.54	6.13 ± 1.78^†^
HDL-cholesterol (mmol/l)	1.35 ± 0.40	1.38 ± 0.41	1.29 ± 0.40*	1.31 ± 0.42^†^	1.28 ± 0.39*
LDL-cholesterol (mmol/l)	4.05 ± 1.41	3.82 ± 1.31	3.92 ± 1.36	3.66 ± 1.24	4.07 ± 1.41^†^
Urinary albumin (mg/l)	13.0 (3.3-149.9)	9.0 (4.9-150.1)	14.5 (3.3-150.1)*	10.5 (4.9-150.0)	17.5 (3.3-150.0)*
***Anthropometric data ***					
Waist-to-hip ratio	0.88 ± 0.08	0.83 ± 0.09	0.91 ± 0.07*	0.89 ± 0.07*	0.92 ± 0.07*
Body mass index (kg/m^2^)	25.8 ± 5.25	24.6 ± 4.9	25.9 ± 5.1*	24.8 ± 5.3	26.5 ± 4.8*
Body fat by BIA (%)^b^	30.2 ± 9.9	28.8 ± 9.6	29.8 ± 10.1^†^	27.9 ± 10.9	30.9 ± 9.4^†^
***History and activity ***					
Diabetes family history (yes, %)	39.9 (585)	26.3 (99)	57.9 (391)*	59.0 (148)*	57.3 (243)*
Hypertension family history (yes, %)	41.5 (608)	30.2 (114)	40.7 (275)*	31.1 (78)	46.5 (197)*
Smoking status (ever, %) ^c^	5.7 (84)	3.7 (14)	7.3 (49)^†^	7.2 (18)	7.3 (31)^†^
Type of main work (light, %)	88.7 (1250)	92.3 (335)	86.1 (558)^†^	83.8 (202)^†^	87.5 (356)^†^
Working time (h/week)	49.3 ± 22.1	48.4 ± 18.3	49.0 ± 24.0	50.0 ± 24.4	48.1 ± 23.6
Recreational sports (yes, %)	21.2 (311)	22.5 (85)	22.2 (150)	19.5 (49)	23.8 (101)
Energy expenditure (MJ/d)	6.30 ± 3.57	5.34 ± 3.00	6.84 ± 3.70*	6.41 ± 3.54*	7.10 ± 3.78*
***Socio-economic data ***					
Literacy (illiterate, %)	35.2 (514)	21.3 (80)	45.8 (308)*	45.0 (113)^†^	46.3 (195)*
Unemployed (%)	26.4 (386)	9.8 (37)	36.9 (248)*	24.7 (62)*	44.2 (186)*
No. of people per household	5 (1-100)	5 (1-70)	6 (1-100)*	6 (1-100)*	6 (1-100)*
Wealth score^d^	0.58 ± 0.17	0.60 ± 0.15	0.57 ± 0.18^†^	0.55 ± 0.18*	0.58 ± 0.18

Values are expressed as means ± standard deviation, median (range) or % (*n*). *, as compared to controls, *P *< 0.001; ^†^, as compared to controls, *P *< 0.05; ^a^, Data of the remaining 414 patients with hypertension only are presented in Additional file 2: Table S2; ^b^, measured by bioelectric impedance analysis; ^c^, includes current and quit smoking; ^d^, proportion positive of 11 markers of wealth: electricity, pipe-borne water, radio, fan, cupboard, television, bicycle, motor-bike, refrigerator, car/truck/tractor, cattle

### Characterisation of diabetic patients

The characteristics of the 675 diabetic patients (mean age, 54.7 years; 75% female) are shown in Table 1 and Additional file 1: Table S1. Almost all DM2 patients (97%) were previously known; 63% additionally had hypertension. Mean FPG was 8.3 ± 4.3 mmol/L, similar in men (9.0 ± 5.2 mmol/L) and women (8.1 ± 3.1 mmol/L; *P *= 0.28). Overweight (53%), obesity (19%), central adiposity (75%), and increased body fat (54%, 56%) were common, and each more frequent in women than men (P < 0.01). Carbohydrates contributed 56 ± 13% to the daily energy intake, protein 19 ± 6%, and fat 33 ± 14%. Reported mean energy expenditure was 6.8 ± 3.7 MJ/d. Serum triglycerides were increased in 31% and total cholesterol in 65%. Cholesterol concentrations were higher in women (6.06 ± 1.69 mmol/L) than men (5.61 ± 1.75 mmol/L; *P *= 0.003). Low HDL-cholesterol characterised 21%.

Increased urinary albumin (≥ 20 mg/L) was measured in 43% of DM2 patients and polyuria was reported by 31%. Almost 20% presented with a history of or clinically assessed complications (peripheral ulcers, 40; diabetic coma, 35; stroke, 25; neuropathy, 13; nephropathy, 5; glaucoma, 5; erectile dysfunction, 5; myocardial infarction, 2; coronary artery disease, 1; retinopathy, 1). Further characteristics are shown in Table 1.

Most DM2 patients (97%) were on antidiabetic medication. The predominant regimes were based on metformin (78%) and sulfonylureas (61%), in addition to glitazones (24%) and insulin (22%, Additional file 3: Table S3). Further medication included lipid lowering drugs (3%), calcium-channel blockers (40%) and angiotensin-converting enzyme inhibitors (33%).

### Univariate associations with DM2

Table 1 displays univariate differences between controls, all DM2 patients, DM2 patients with and without hypertension, and patients with hypertension only. Analysis stratified by gender is shown in Additional file 1: Table S1.

In comparison with controls (each, *P *< 0.05), the 675 DM2 patients were older, had higher WHR, BMI, percentage body fat, BP, triglycerides, and urinary albumin as well as reduced HDL-cholesterol. Twice as many DM2 patients as controls reported a family history of diabetes; a family history of hypertension was also more frequent. More DM2 patients than controls resided in Kumasi outskirts, and in larger households with fewer assets, were illiterate or unemployed, and smokers. Patients less frequently than controls pursued light work and thus had a higher daily energy expenditure. Fat and salt intakes were increased in DM2 patients as compared to controls (means ± SD, fat, 36 ± 14 *vs*. 33 ± 12 g/4.2 MJ/d, *P *< 0.001; sodium, 3.4 ± 1.9 *vs*. 3.2 ± 1.8 g/d, *P *< 0.001). No further significant differences were observed.

Stratifying DM2 patients into those with and without hypertension basically confirmed the above differences as compared to controls (Table 1). However, hypertensive DM2 patients presented with the worst lipid profile, the greatest measures of obesity and the highest urinary albumin. SES and activity level were lowest among DM2 patients with hypertension. Also, they had the highest proportion of smokers and hypertension family history. Nutritional values were similar to the non-hypertensive DM2 group.

As for patients with hypertension only, they were older and more obese than controls. FPG, triglycerides, total cholesterol and urinary albumin were significantly increased. Compared to controls, they more frequently had a family history of hypertension, lived in Kumasi outskirts, were illiterate and unemployed. Activity level and nutritional behaviour, however, were similar in this and the control group (Additional file 2: Table S2).

### Multivariate associations with DM2

Factors independently associated with DM2 were identified in a logistic regression model (age and gender *a priori *included) following a stepwise backward approach: all univariately associated factors were included in the model and non-associated variables (*P *> 0.05) were consecutively removed (Table 2). A family history of diabetes (aOR, 3.8) and abdominal adiposity (aOR, 2.6) were the most strongly, independently associated factors. Other independently associated parameters increasing the odds of DM2 were unemployment, crowded living conditions, extended working time, illiteracy, outskirts residence, increased triglycerides, and < 55% contribution of carbohydrates to the daily energy intake. In a fully adjusted model, these findings were basically confirmed (Table 2).

**Table 2:** Factors associated with diabetes mellitus type 2 in multivariate analysis

Parameter	aOR for diabetes (95% CI)	aOR for diabetes without hypertension (95% CI)	aOR for diabetes with hypertension (95% CI)
Residence (Kumasi outskirts)	1.93 (1.27-2.93)	1.78 (1.12-2.84)	1.96 (1.16-3.31)
Triglycerides ≥ 1.695 mmol/l	1.83 (1.13-2.97)	-	2.46 (1.40-4.32)
Increased waist-to-hip ratio ^a^	2.63 (1.76-3.93)	2.92 (1.85-4.61)	2.93 (1.79-4.81)
Diabetes family history, positive	3.79 (2.60-5.51)	3.92 (2.56-6.01)	3.72 (2.35-5.88)
Type of main work (light)	0.44 (0.25-0.78)	0.50 (0.26-0.96)	0.30 (0.15-0.59)
Working time > 40 h/week	1.76 (1.20-2.57)	1.85 (1.19-2.87)	1.84 (1.15-2.95)
Illiteracy	1.95 (1.28-2.97)	2.27 (1.43-3.62)	-
Unemployment	4.23 (2.33-7.65)	2.58 (1.30-5.12)	5.71 (2.95-11.06)
Crowded living condition ^b^	2.78 (1.71-4.51)	2.59 (1.53-4.40)	3.58 (1.97-6.48)
Carbohydrate < 55% of energy	1.63 (1.12-2.35)	-	2.02 (1.27-3.22)

aOR, adjusted odds ratio from multivariate logistic regression. Age and gender were a priori included. All univariately associated variables were included and stepwise backward removal of insignificantly associated factors (*P *> 0.05) identified independently associated parameters. R^2 ^for diabetes, 0.53; R^2 ^for diabetes without hypertension, 0.39; R^2 ^for diabetes with hypertension, 0.65. All univariately associated parameters from Table 1 (partially dichotomised) were included in a fully adjusted model. The type and degree of associations held true; each aOR changed < 44%. ^a^, male, ≥ 0.90; female, ≥ 0.85; ^b^, > 75^th ^percentile of the number of people per household (*n *> 8)

Following stratification by hypertension, the risk estimates as mentioned above remained basically the same (Table 2), with a few exemptions: At concomitant hypertension, the associations with DM2 strengthened for increased triglycerides and low carbohydrate contribution to energy intake. In contrast, these two factors were not associated with DM2 in the absence of hypertension (Table 2). Exclusive hypertension was independently associated with a respective family history, central adiposity, high triglycerides, and extended working time (Additional file 2: Table S2).

## Discussion

DM2 is emerging literally epidemically in SSA [3]. This hospital-based study from urban Ghana shows that DM2 patients are predominantly middle-aged and of low socio-economic status, and characterized by high proportions of central adiposity, a respective family history, hypertension, albuminuria and hyperlipidaemia.

The study population - although not representative for the community as a whole - displays many features of urban life in Africa: life-style is mainly sedentary, socio-economic conditions are severely restricted, overweight is frequent, particularly among women, alcohol and tobacco use are generally low, and recreational sports not very popular [21-25]. In comparison with previous studies on DM2 in urban Africa, our findings on obesity and SES are similar. However, our diabetic patients exhibited lower FPG and blood pressure, worse lipid profiles, more frequently a family history of DM2, and less use of tobacco and alcohol [10,23,26-28]. In comparison to African American DM2 patients, blood pressure and lipid profiles were similar in the present study but obesity, family history, and smoking and alcohol intake less frequent [21,29]. In comparison to African and African American DM2 patients, Caucasian DM2 patients show the worst lipid profiles, the highest rates of tobacco and alcohol consumption, a male predominance in abdominal adiposity, and more physical activity [21,30,31].

Rates of hypertension and albuminuria were high among our DM2 patients, confirming previous findings from Kumasi [32]. This suboptimal management of patients may reflect both institutional and individual factors including drug cost and availability, health policy disparities, culturally inappropriate lifestyle recommendations, and diluting effects of traditional medicine [33]. Still, complications occurred at only half the figure reported elsewhere in SSA [28,34]. Diagnostic restrictions and a majority of medicated patients may be involved. Typically, in African diabetic patients, late-onset microvascular complications predominate over macrovascular events [6,34]. In fact, reported rates of retinopathy in African DM2 patients (25%) exceed those in African Americans (10%) and Caucasians (9%), whereas cardiovascular diseases are estimated at 8%, 33% and 48%, respectively [34,35].

Limitations of the study and of associated factor analysis in particular need to be considered. Our DM2 definition by FPG is based on the IDF consensus valid during study conduct and follows general practice. Validated nutritional questionnaires possibly could have improved respective assessments. Importantly, the present study was not matched for e.g., age and sex, and used a convenience sample. Women predominated. Controls were younger than patients and, related to that, roughly a quarter originated from hospital staff. This basically was due to limited project funds and reluctance of community members to participate. Although the multivariate analyses are adjusted for these and other differences, the selection bias has implications for interpreting the results: for instance, the increased odds of DM2 among unemployed individuals are partially an artefact due to the proportion of staff members among controls. This, however, does not invalidate the identification of major risk factors such as, e.g., family history or abdominal adiposity. In analysis, we followed an exploratory approach, i.e., lacking pre-formulated hypotheses, and identified independently associated factors. Thus, associations of e.g. unemployment or crowded living conditions with DM2 are statistically independent which does not mean that they are unrelated in real life. We stratified analysis by the presence of hypertension to illustrate differences in associated factors. For comprehensibility, we abstained from (further) stratification by e.g. age groups but adjusted for age, sex and obesity. Lastly and as a matter of fact, association does not necessarily mean causality, and the direction of an association is subject to interpretation.

Notwithstanding the above mentioned limitations, several and partly inter-related parameters reflecting low SES strongly associated with DM2. The propagation of DM2 among low social classes worldwide may be due to low health care utilization, reduced uptake of prevention messages and SES-dependent differences in risk factors including nutrition and physical inactivity [36]. Associations of DM2 with outskirts residence and illiteracy point to the possibility of inadequate access to health information. Unemployment, expanded working hours, hard work, and overcrowded households were all associated with DM2 and may reflect stressful living conditions. Psycho-social stressors are known to be capable of adversely influencing the metabolic constitution [37]. Stress may lead to overeating and poor exercise. Also, increased sympathetic activity may affect adipose and pancreatic tissue regulation and contribute to insulin resistance [38]. Detailed investigations into the association of psycho-social stress and DM2 in SSA are thus warranted.

The strong association in the present study of DM2 with a respective family history underlines the pronounced predisposition in Africans towards DM2 [39]. However, replication of risk alleles established in Caucasians not rarely has failed in African populations [40], possibly as a result of their higher genetic diversity [41]. Because of this, validated genetic markers of an increased risk of DM2 in Africans are rare. Large-scale studies accounting for environmental variation and, possibly, epigenetic priming, will thus be needed to disentangle predisposition in, e.g. the Ghanaian population.

Obesity, a prominent DM2 risk factor worldwide [42] and also in the present study, shows an outstanding prevalence in SSA, particularly in urban women [22]. In many areas of SSA, obesity constitutes an obvious social marker of affluence, and poor knowledge and misconceptions about lifestyle risk factors conflict with appropriate prevention and control of obesity and DM2. Clearly, more research into the traditional cognitive imagery as well as into DM2-related knowledge, attitudes and behaviour is needed to be able to implement socioculturally appropriate health promotion campaigns [1,43]. Such is of particular importance considering the specifically increased risk for adulthood obesity (and DM2) as a result of frequent undernutrition in African infants [44].

Serum lipid profiles are constantly associated with DM2 [30], and so did hyperlipidaemia in the present study, particularly when DM2 was complicated by hypertension. Hyperlipidaemia may result from a combination of low SES and comparatively high urban food prices which, in turn, favours the intake of inexpensive and highly refined foods, i.e. poor in fibre and protein, but rich in simple carbohydrates, fats and sodium [10,44]. In fact, such corresponds to the diet assessed for most study participants. Contrariwise, popular meals based on peanut and fermented maize may improve lipid profiles and underlie the observed weak association of DM2 with total and HDL-cholesterol [45,46].

## Conclusions

In this study from urban Ghana, DM2 was predominately observed among individuals of rather low socio-economic status contrasting with the still prevalent perception of DM2 as a disease of affluence. High rates of hypertension and albuminuria among the largely pre-diagnosed DM2 patients point to the necessity of improved management. The associations of DM2 with factors related to low socio-economic status and/or psycho-social stress indicate a specific pattern of DM2 risks in this population. For immediate impact, improved management of complications, access to early diagnosis and treatment, and health worker training appear to be vital. For primary prevention of DM2 in this population, the verification of associated factors by longitudinal studies is warranted.

## Competing interests

The authors declare that they have no competing interests.

## Authors' contributions

ID, GBA, MvdG, JS and FPM conceived and designed the study. ID, GBA, KJT, FM, YAAm, and YAAw were responsible for recruitment, interviews and examinations of study participants. JS did the lipid analysis. ID, ED, and FPM performed the statistical analysis. ID and FPM wrote the manuscript with contributions of all authors. All authors read and approved the final manuscript.

## Pre-publication history

The pre-publication history for this paper can be accessed here:

http://www.biomedcentral.com/1471-2458/12/210/prepub

## Supplementary Material

Additional file 1 **Table S1**. Characteristics of 1466 urban Ghanaians with and without type 2 diabetes mellitus and/or hypertension stratified by gender. Click here for file

Additional file 2 **Table S2**. Characteristics of 791 urban Ghanaians with and without hypertension stratified by gender. Click here for file

Additional file 3 **Table S3**. Antidiabetic medication use among 675 patients with diabetes mellitus type 2 in Ghana. Click here for file
