# Supplementary material for: Diabetes mellitus type 2 in urban Ghana: characteristics and associated factors
Source: BMC Public Health. 2012 Mar 20;12:210. doi: 10.1186/1471-2458-12-210 (PMC3364878; doi:10.1186/1471-2458-12-210)
Supplement: Additional file 1 — Table S1. Characteristics of 1466 urban Ghanaians with and without type 2 diabetes mellitus and/or hypertension stratified by gender. [file 1471-2458-12-210-S1.DOC]

#### Supplementary Table 1 - Characteristics of 1466 urban Ghanaians with and without type 2 diabetes mellitus and/or hypertension stratified by gender

| **Characteristics** | **Controls**  **(*n* = 377)** | |  | **Diabetes mellitus type 2 (DM2)**  **(*n* = 675)** | |  | **DM2 without hypertension**  **(*n* = 251)** | |
| --- | --- | --- | --- | --- | --- | --- | --- | --- |
|  | male | female |  | male | female |  | male | female |
| *n* | 89 | 288 |  | 172 | 503 |  | 71 | 180 |
| Age (years) | 37.7 ± 13.3 | 39.1 ± 15.3 |  | 55.1± 14.1* | 54.6 ± 13.1* |  | 49.8 ± 15.0* | 47.9 ± 14.1* |
| Ethnic group (Akan, %) | 83.1 (74) | 84.7 (244) |  | 88.4 (152)* | 87.6 (441)* |  | 87.3 (62)* | 82.1 (148)*^,†^ |
| Residence (Kumasi metropolitan, %) | 82.0 (73) | 78.5 (266) |  | 65.7 (113)* | 72.6 (365)* |  | 59.2 (42)* | 72.5 (131)* |
| **Clinical data** |  |  |  |  |  |  |  |  |
| Fasting plasma glucose (mmol/l) | 4.53 ± 0.78 | 4.51 ± 0.62 |  | 9.02 ± 5.20* | 8.07 ± 3.96* |  | 9.75 ± 5.21* | 8.62 ± 4.22* |
| Systolic blood pressure (mmHg) | 119.3 ± 11.1 | 114.9 ± 11.2 |  | 137.7 ± 22.5* | 138.6 ± 24.4* |  | 120.0 ± 11.6 | 118.1 ± 13.0* |
| Diastolic blood pressure (mmHg) | 75.8 ± 7.4 | 76.0 ± 7.5 |  | 84.2 ± 13.0* | 85.5 ± 11.7* |  | 75.3 ± 8.3 | 77.0 ± 7.0* |
| Triglycerides (mmol/l) | 1.12 ± 0.56 | 1.15 ± 0.56 |  | 1.47 ± 0.92* | 1.58 ± 0.77* |  | 1.36 ± 0.62* | 1.41 ± 0.64* |
| Total cholesterol (mmol/l) | 5.29 ± 1.54 | 5.87 ± 1.61 |  | 5.61 ± 1.75* | 6.06 ± 1.69* |  | 5.50 ± 1.69* | 5.86 ± 1.49 |
| HDL-cholesterol (mmol/l) | 1.27 ± 0.39 | 1.42 ± 0.41 |  | 1.23 ± 0.42* | 1.31 ± 0.39* |  | 1.26 ± 0.44 | 1.30 ± 0.41* |
| LDL-cholesterol (mmol/l) | 3.50 ± 1.21 | 3.92 ± 1.33 |  | 3.69 ± 1.36* | 4.00 ± 1.35* |  | 3.59 ± 1.38 | 3.69 ± 1.18* |
| Urinary albumin (mg/l) | 8 (0-145) | 9 (0-145) |  | 17 (0-145)* | 13 (0-147)* |  | 11 (0-145)* | 10 (0-145)* |
| **Anthropometric data** |  |  |  |  |  |  |  |  |
| Waist-to-hip ratio | 0.85 ± 0.06 | 0.83 ± 0.10 |  | 0.92 ± 0.06* | 0.91 ± 0.07* |  | 0.91 ± 0.06* | 0.89 ± 0.08* |
| Body mass index (kg/m^2^) | 22.5 ± 3.3 | 25.2 ± 5.2 |  | 23.3 ± 3.7* | 26.8 ± 5.1* |  | 22.1 ± 3.4* | 25.8 ± 5.6* |
| Body fat by BIA (%) ^a^ | 18.2 ± 6.2 | 32.1 ± 7.9 |  | 18.5 ± 7.2 | 33.6 ± 7.7* |  | 17.4 ± 7.3* | 32.1 ± 9.1 |
| **History and activity** |  |  |  |  |  |  |  |  |
| Diabetes family history (yes, %) | 20.2 (18) | 28.1 (81) |  | 52.9 (91)* | 68.6 (345)*^,†^ |  | 59.2 (42)* | 68.9 (124)*^,†^ |
| Hypertension family history (yes, %) | 15.7 (14) | 34.7 (100)^†^ |  | 36.0 (62)* | 58.4 (294)*^,†^ |  | 35.2 (25)* | 49.4 (89)* |
| Smoking status (ever, %) ^b^ | 14.6 (13) | 0.3 (1)^†^ |  | 24.4 (42)* | 1.4 (7)*^,†^ |  | 23.9 (17)* | 0.6 (1)^†^ |
| Type of main work (light, %) | 84.7 (75) | 94.6 (272)^†^ |  | 77.1 (133)* | 89.2 (449)*^,†^ |  | 75.0 (53)* | 87.3 (157)*^,†^ |
| Working time (h/week) | 51.8 ± 21.5 | 47.6 ± 19.6 |  | 59.2 ± 31.3 | 60.5 ± 32.7 |  | 52.6 ± 25.4 | 56.8 ± 30.5 |
| Recreational sports (yes, %) | 41.6 (37) | 16.7 (48)^†^ |  | 33.2 (57) | 18.5 (93)^†^ |  | 31.0 (22) | 15.0 (27)^†^ |
| Energy expenditure (MJ/d) | 6.30 ± 3.66 | 5.04 ± 2.70 |  | 7.82 ± 4.51* | 6.48 ± 3.30* |  | 7.18 ± 4.29* | 6.11 ± 3.16* |
| **Socio-economic data** |  |  |  |  |  |  |  |  |
| Literacy (illiterate, %) | 13.5 (12) | 23.7 (68)^†^ |  | 20.5 (35)* | 54.5 (274)*^,†^ |  | 23.9 (17)* | 53.4 (96)*^,†^ |
| Occupation (unemployed, %) | 6.7 (6) | 10.8 (31) |  | 28.7 (49)* | 39.7 (200)*^,†^ |  | 16.9 (12)* | 27.8 (50)* |
| No. of people per household | 5 (1-70) | 5 (1-50) |  | 6 (1-50)* | 6 (1-100)* |  | 6 (1-40)* | 6 (1-100)* |
| Wealth score ^c^ | 0.60 ± 0.14 | 0.60 ± 0.15 |  | 0.58 ± 0.18 | 0.56 ± 0.18* |  | 0.57 ± 0.16* | 0.54 ± 0.19* |

**Table Legend.**

Values are expressed as means ± standard deviation, median (range) or % (n). *, as compared to controls, *P* < 0.05; ^†^, differences between men and women of same stratum, *P*<0.05; ^a^, measured by bioelectric impedance analysis; ^b^, includes current and quit smoking; ^c^, proportion positive of 11 markers of wealth: electricity, pipe-borne water, radio, fan, cupboard, television, bicycle, motor-bike, refrigerator, car/truck/tractor, cattle

The following parameters differed significantly (*P* < 0.05) between **controls and all diabetic patients** in age-adjusted logistic regression (binary variable) or quantile regression (numeric variable): residence, fasting plasma glucose, systolic blood pressure, diastolic blood pressure, triglycerides, HDL-cholesterol, urinary albumin, waist-to-hip ratio, diabetes family history, hypertension family history, type of main work, working time, energy expenditure, literacy, occupation, no. of people per household.

The following parameters differed significantly (*P* < 0.05) between **controls and diabetic patients without hypertension** in age-adjusted logistic regression (binary variable) or quantile regression (numeric variable): residence, fasting plasma glucose, triglycerides, total cholesterol, LDL-cholesterol, waist-to-hip ratio, diabetes family history, type of main work, energy expenditure, occupation, no. of people per household.
