# Supplementary material for: Diabetes mellitus type 2 in urban Ghana: characteristics and associated factors
Source: BMC Public Health. 2012 Mar 20;12:210. doi: 10.1186/1471-2458-12-210 (PMC3364878; doi:10.1186/1471-2458-12-210)
Supplement: Additional file 2 — Table S2. Characteristics of 791 urban Ghanaians with and without hypertension stratified by gender. [file 1471-2458-12-210-S2.DOC]

#### Supplementary Table 2 - Characteristics of 791 urban Ghanaians with and without hypertension stratified by gender

| Characteristics | **Controls** | | |  | **Hypertension only** | | |
| --- | --- | --- | --- | --- | --- | --- | --- |
|  | total | male | female |  | total | male | female |
| n | 377 | 89 | 288 |  | 414 | 91 | 323 |
| Age (years) | 38.8 ± 14.8 | 37.7 ± 13.3 | 39.1 ± 15.3 |  | 54.7 ± 12.7* | 55.0 ± 13.8* | 54.7 ± 12.4* |
| Ethnic group (Akan, %) | 84.4 (318) | 83.1 (74) | 84.7 (244) |  | 88.6 (367) | 89.2 (81)* | 84.0 (271) |
| Residence (Kumasi metropolitan, %) | 79.3 (299) | 82.0 (73) | 78.5 (266) |  | 73.4 (304)* | 74.2 (68)* | 73.2 (236)* |
| Clinical *data* |  |  |  |  |  |  |  |
| Fasting plasma glucose (mmol/l) | 4.5 ± 0.7 | 4.53 ± 0.78 | 4.51 ± 0.62 |  | 4.6 ± 0.8* | 4.59 ± 0.86* | 4.55 ± 0.71* |
| Systolic blood pressure (mmHg) | 116.0 ± 11.3 | 119.3 ± 11.1 | 114.9 ± 11.2 |  | 144.8 ± 20.4* | 150.8 ± 20.8* | 143.0 ± 20.0* |
| Diastolic blood pressure (mmHg) | 76.0 ± 7.5 | 75.8 ± 7.4 | 76.0 ± 7.5 |  | 92.2 ± 12.3* | 94.0 ± 14.3* | 91.7 ± 11.6* |
| Triglycerides (mmol/l) | 1.15 ± 0.56 | 1.12 ± 0.56 | 1.15 ± 0.56 |  | 1.53 ± 0.70* | 1.50 ± 0.72* | 1.54 ± 0.70* |
| Total cholesterol (mmol/l) | 5.73 ± 1.61 | 5.29 ± 1.54 | 5.87 ± 1.61 |  | 6.62 ± 1.81* | 6.19 ± 1.80* | 6.74 ± 1.79* |
| HDL-cholesterol (mmol/l) | 1.38 ± 0.41 | 1.27 ± 0.39 | 1.42 ± 0.41 |  | 1.40 ± 0.38 | 1.33 ± 0.39* | 1.43 ± 0.38 |
| LDL-cholesterol (mmol/l) | 3.82 ± 1.31 | 3.50 ± 1.21 | 3.92 ± 1.33 |  | 4.47 ± 1.48* | 4.15 ± 1.38* | 4.57 ± 1.50* |
| Urinary albumin (mg/l) | 9.0 (4.9-150) | 8 (0-145) | 9 (0-145) |  | 16.0 (4.9-149)* | 18 (0-145)* | 16 (0-145)* |
| Anthropometric *data* |  |  |  |  |  |  |  |
| Waist-to-hip ratio | 0.83 ± 0.09 | 0.85 ± 0.06 | 0.83 ± 0.10 |  | 0.89 ± 0.07* | 0.92 ± 0.08* | 0.88 ± 0.07* |
| Body mass index (kg/m^2^) | 24.6 ± 4.9 | 22.5 ± 3.3 | 25.2 ± 5.2 |  | 26.9 ± 5.6* | 24.2 ± 4.3* | 27.7 ± 5.7* |
| Body fat by BIA (%) ^a^ | 28.8 ± 9.6 | 18.2 ± 6.2 | 32.1 ± 7.9 |  | 32.3 ± 9.5* | 21.2 ± 6.6* | 35.5 ± 7.6* |
| *History and activity* |  |  |  |  |  |  |  |
| Diabetes family history (yes, %) | 26.3 (99) | 20.2 (18) | 28.1 (81) |  | 22.9 (95) | 26.9 (25)* | 30.2 (98) |
| Hypertension family history (yes, %) | 30.2 (114) | 15.7 (14) | 34.7 (100)^†^ |  | 52.9 (219)* | 52.7 (48)* | 61.4 (198)*^,†^ |
| Smoking status (ever, %) ^b^ | 3.7 (14) | 14.6 (13) | 0.3 (1)^†^ |  | 5.1 (21) | 20.7 (19) | 0.6 (2)^†^ |
| Type of main work (light, %) | 92.3 (335) | 84.7 (75) | 94.6 (272)^†^ |  | 89.5 (358) | 85.2 (78) | 90.7 (293)* |
| Working time (h/week) | 48.4 ± 18.3 | 51.8 ± 21.5 | 47.6 ± 19.6 |  | 50.8 ± 23.5 | 55.9 ± 30.0 | 55.7 ± 31.2 |
| Recreational sports (yes, %) | 22.5 (85) | 41.6 (37) | 16.7 (48)^†^ |  | 18.4 (76) | 33.0 (30) | 14.2 (46)^†^ |
| Energy expenditure (MJ/d) | 5.34 ± 3.00 | 6.30 ± 3.66 | 5.04 ± 2.70 |  | 6.35 ± 3.67* | 6.74 ± 3.76* | 6.24 ± 3.64* |
| *Socio-*economic *data* |  |  |  |  |  |  |  |
| Literacy (illiterate, %) | 21.3 (80) | 13.5 (12) | 23.7 (68)^†^ |  | 30.5 (126)* | 8.7 (8)* | 36.8 (119)*^,†^ |
| Unemployed (%) | 9.8 (37) | 6.7 (6) | 10.8 (31) |  | 24.5 (101)^†^ | 20.0 (18)* | 25.8 (83)* |
| No. of people per household | 5 (1-70) | 5 (1-70) | 5 (1-50) |  | 5 (1-55) | 5 (1-29) | 5 (1-55) |
| Wealth score ^c^ | 0.60 ± 0.15 | 0.60 ± 0.14 | 0.60 ± 0.15 |  | 0.58 ± 0.18 | 0.61 ± 0.15 | 0.57 ± 0.18* |

**Table Legend.**

Values are expressed as means ± standard deviation, median (range) or % (*n*). *, as compared to controls, *P*<0.001; ^†^, as compared to controls, *P*<0.05; ^a^, measured by bioelectric impedance analysis; ^b^, includes current and quit smoking; ^c^, proportion positive of 11 markers of wealth: electricity, pipe-borne water, radio, fan, cupboard, television, bicycle, motor-bike, refrigerator, car/truck/tractor, cattle

The following parameters differed significantly (*P* < 0.05) between **controls and hypertensive patients** in age-adjusted logistic regression (binary variable) or quantile regression (numeric variable): systolic blood pressure, diastolic blood pressure, triglycerides, total cholesterol, LDL-cholesterol, urinary albumin, waist-to-hip ratio, body mass index, body fat, hypertension family history, energy expenditure, literacy, occupation.

Age and gender were a priori included in a multivariate model with all univariately associated variables. A stepwise backward removal of insignificantly associated factors (*P*>0.05) identified independently associated parameters (age-adjusted odds ratio, (95% confidence interval)): triglycerides ≥1.695 mmol/l, 1.93 (1.22-3.03); Increased waist-to-hip ratio (male, ≥0.90; female, ≥0.85), 1.94 (1.32-2.86); Hypertension family history, positive, 2.49 (1.72-3.61); Working time >40 h/week, 1.59 (1.09-2.33); R^2^ for hypertension, 0.42. All univariately associated parameters (partially dichotomised) were included in a fully adjusted model. The type and degree of associations held true; each aOR changed <44%.
