# Supplementary material for: Diabetes mellitus type 2 in urban Ghana: characteristics and associated factors
Source: BMC Public Health. 2012 Mar 20;12:210. doi: 10.1186/1471-2458-12-210 (PMC3364878; doi:10.1186/1471-2458-12-210)
Supplement: Additional file 3 — Table S3. Antidiabetic medication use among 675 patients with diabetes mellitus type 2 in Ghana. [file 1471-2458-12-210-S3.DOC]

## Supplementary Table 3.

## Antidiabetic medication use among 675 patients with diabetes mellitus type 2 in Ghana

| **Medication** |  | ***n*** |  | **%** |
| --- | --- | --- | --- | --- |
| Any antidiabetic |  | 651 |  | 96.4 |
| Monotherapy |  | 149 |  | 22.1 |
| Metformin |  | 54 |  | 8.0 |
| Sulfonylureas |  | 50 |  | 7.4 |
| Glitazones |  | 4 |  | 0.6 |
| Insulins |  | 32 |  | 4.7 |
| Others ^a^ |  | 9 |  | 1.3 |
| Double therapy |  | 381 |  | 56.4 |
| Metformin + sulfonylureas |  | 250 |  | 37.0 |
| Metformin + insulins |  | 81 |  | 12.0 |
| Metformin + glitazones |  | 22 |  | 3.3 |
| Sulfonylureas + glitazones |  | 19 |  | 2.8 |
| Glitazones + insulins |  | 7 |  | 1.0 |
| Sulfonylureas + insulins |  | 2 |  | 0.3 |
| Others ^a^ |  | 10 |  | 1.5 |
| Triple therapy |  | 111 |  | 16.4 |
| Metformin + sulfonylureas + glitazones |  | 86 |  | 12.7 |
| Metformin + insulins + glitazones |  | 22 |  | 3.3 |
| Metformin + sulfonylureas + insulins |  | 3 |  | 0.4 |

^a^, include dietary intervention, lipid lowering drugs and others
